# Silencing of the Rotavirus NSP4 Protein Decreases the Incidence of Biliary Atresia in Murine Model

**DOI:** 10.1371/journal.pone.0023655

**Published:** 2011-08-18

**Authors:** Jiexiong Feng, Jixin Yang, Shuaiyu Zheng, Yinrong Qiu, Chengwei Chai

**Affiliations:** Department of Pediatric Surgery, Tongji Hospital, Tongji Medical College, Huazhong University of Science and Technology, Wuhan, China; Institut Pasteur, France

## Abstract

Biliary atresia is a common disease in neonates which causes obstructive jaundice and progressive hepatic fibrosis. Our previous studies indicate that rotavirus infection is an initiator in the pathogenesis of experimental biliary atresia (BA) through the induction of increased nuclear factor-kappaB and abnormal activation of the osteopontin inflammation pathway. In the setting of rotavirus infection, rotavirus nonstructural protein 4 (NSP4) serves as an important immunogen, viral protein 7 (VP7) is necessary in rotavirus maturity and viral protein 4 (VP4) is a virulence determiner. The purpose of the current study is to clarify the roles of NSP4, VP7 and VP4 in the pathogenesis of experimental BA. Primary cultured extrahepatic biliary epithelia were infected with Rotavirus (mmu18006). Small interfering RNA targeting NSP4, VP7 or VP4 was transfected before rotavirus infection both in vitro and in vivo. We analyzed the incidence of BA, morphological change, morphogenesis of viral particles and viral mRNA and protein expression. The in vitro experiments showed NSP4 silencing decreased the levels of VP7 and VP4, reduced viral particles and decreased cytopathic effect. NSP4-positive cells had strongly positive expression of integrin subunit α2. Silencing of VP7 or VP4 partially decreased epithelial injury. Animal experiments indicated after NSP4 silencing, mouse pups had lower incidence of BA than after VP7 or VP4 silencing. However, 33.3% of VP4-silenced pups (N = 6) suffered BA and 50% of pups (N = 6) suffered biliary injury after VP7 silencing. Hepatic injury was decreased after NSP4 or VP4 silencing. Neither VP4 nor VP7 were detected in the biliary ducts after NSP4. All together, NSP4 silencing down-regulates VP7 and VP4, resulting in decreased incidence of BA.

## Introduction

Biliary atresia (BA) is a common biliary disease in infants. It is characterized by progressive destruction of extrahepatic bile ducts, resulting in obstruction of bile flow during the first few months of children's lives [1], [2]. Interdisciplinary initiatives [3] focusing on BA suggests it is a virus-induced autoimmune disease [4], [5]. As supported by our clinical evidence [6], [7], [8] and animal models [9], [10], reovirus, especially rotavirus, infection could cause biliary injury or obstruction of bile duct lumens. Among all candidates, rhesus rotavirus (RRV) is considered to be the most potent virus in inducing experimental BA [9]. Nonstructural protein 4 (NSP4), viral protein 7 (VP7) and viral protein 4 (VP4) have become hotspots for the mechanism of rotavirus infection as they are important in the process of rotavirus replication [11], [12], [13]. However, studies investigating the relationship between rotavirus proteins and BA are lacking.

Besides the important role in the replication process of rotavirus, NSP4 serves as a potential immunogen and an enterotoxin [5], VP7 is necessary in rotavirus maturity [12] and VP4 is thought to be a virulence determiner [11]. However, it is unclear what roles they play in the pathogenesis of BA.

Since the utilization of small interfering RNA (siRNA), siRNAs have been used as efficient and powerful tools to “silence” rotavirus genes [12]. In this study, rotavirus NSP4, VP7 and VP4 mRNAs are silenced in biliary epithelia. The roles of NSP4, VP7 and VP4 in the pathogenesis of virus-induced BA are investigated, both in cultured cells and mucosal layers of bile ducts. Our hypothesis is that the loss of function of these key genes can prevent rotavirus associated biliary atresia in rotavirus infected mice.

## Results

### In vitro studies

#### Cell injury, number of viral particles and maturity of rotavirus is decreased by siRNAs

**Figure 1:**
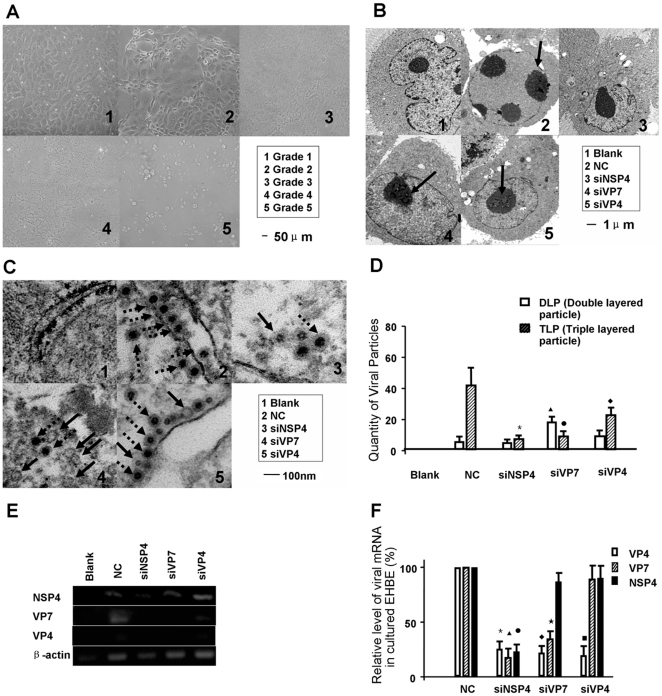
Rotavirus replication, maturity and cytopathic effect of cultured extrahepatic biliary epithelia (EHBE). (A) Cytopathic effect (CPE) grading of cultured EHBE. The severity of EHBE injury was graded from 0 to 4 with 4 being the most severe. (B) Ultrastructural CPE. siNSP4 transfected EHBE had normal structure. The other 2 siRNAs also protected the shape of EHBE, but nuclear degeneration was noted (pointed by black arrows). (C) Rotavirus particles. Significantly less double-layered particles (DLPs, pointed by black arrows) and triple-layered particles (TLPs, pointed by black arrow heads with dash lines) existed in siNSP4 protected EHBE. siVP7 caused more synthesis of DLPs. (D) Viral particle quantification. TLPs in EHBE significantly reduced in all siRNA trasfected groups (^*•♦^ *P*<0.05, compared to NC). DLPs in siVP7 group was increased significantly (^▴^ *P*<0.05, compared to NC). (E) Representative gel images of reverse transcription polymerase chain reaction (RT-PCR). RT-PCR revealed the presence of 3 viral messenger RNAs (mRNAs) in NC group, but siNSP4 transfection reduced the level of NSP4 mRNA and completely inhibited the transcription of VP7 and VP4 mRNA. siVP7 caused absence of VP4 and VP7 mRNA. siVP4 reduced mRNA transcription. (F) Quantitative analysis using Gel Pro Analyzer. siNSP4 decreased the relative level of all viral mRNAs (^*▴•^ *P*<0.05, compared to NC). siVP7 inhibited the transcription of VP7 and VP4 mRNAs (^♦★^ *P*<0.05, compared to NC). siVP4 only inhibited the mRNA expression of VP4 (^▪^ *P*<0.05, compared to NC).

We first examined the cytopathic effect (CPE) of cultured extrahepatic biliary epithelia (EHBE) according to Jafri M's method [14]. CPE was graded according to the CPE percentage: Grade 0: no CPE; Grade 1: 1–25%; Grade 2: 25–50%; Grade 3: 50–75%; Grade 4: 75–100% (Figure 1A). As shown in Table 1, no significant CPE was noted in the blank control group. siNSP4 treated EHBE had the lowest CPE percentage.(*P*<0.05, compared to the negative control group, siVP7 group and siVP4 group), while there was no difference between siVP7 treated, siVP4 treated and negative control EBHE. In addition, transmission electronic microscopy showed siRNA targeting NSP4 (siNSP4) transfected EHBE preserved the normal untrastructure (Figure 1B). siNSP4 also decreased the number of both mature virus (triple layered particles, TLPs) and immature virus (double layered particles, DLPs) (both *P*<0.05, compared to NC). siRNA targeting VP7 (siVP7) transfected EHBE contained more DLPs but less TLPs (both *P*<0.05 compared to NC); siVP4 treated EHBE contained less TLPs, but there was no difference of the number of DLPs in this group compared to the negative control (Figure 1C and 1D).

**Table 1:** Mean CPE percentage of different groups.

	Blank Control^a^ (%)	Negative Control^b^ (%)	siNSP4^c^ (%)	siVP7^d^ (%)	siVP4^e^ (%)
Mean CPE percentage	12.28±3.12	69.39±10.17^#^	26.72±7.63^*^	50.56±9.86^▴^	53.50±9.98^•^

a Blank Control, cells in blank control group that were not infected with rotavirus but transfected with siRNA Lamin A/C.

b Negative Control, cells in negative control were infected with rotavirus and transfected with siRNA Lamin A/C.

c, d, e Cells in these groups were transfected with corresponding siRNAs.

#### Viral mRNA transcription is decreased by siRNAs

RT-PCR showed that the amplicon of VP4, VP7 could not be detected and relatively less NSP4 transcription was noted in siNSP4 transfected EHBE. VP4 and VP7 mRNA was diminished in siVP7 transfected EHBE, but NSP4 mRNA appeared in this group. Decreased transcription level of VP4 was noted in siVP4 transfected EHBE, but the mRNAs of VP7 and NSP4 were both present. The statistical significance of these findings was analyzed using Gel-Pro Analyzer (Version 3.0). (Figure 1E and 1F).

#### Translated products of rotavirus protein are diminished by siRNAs

**Figure 2:**
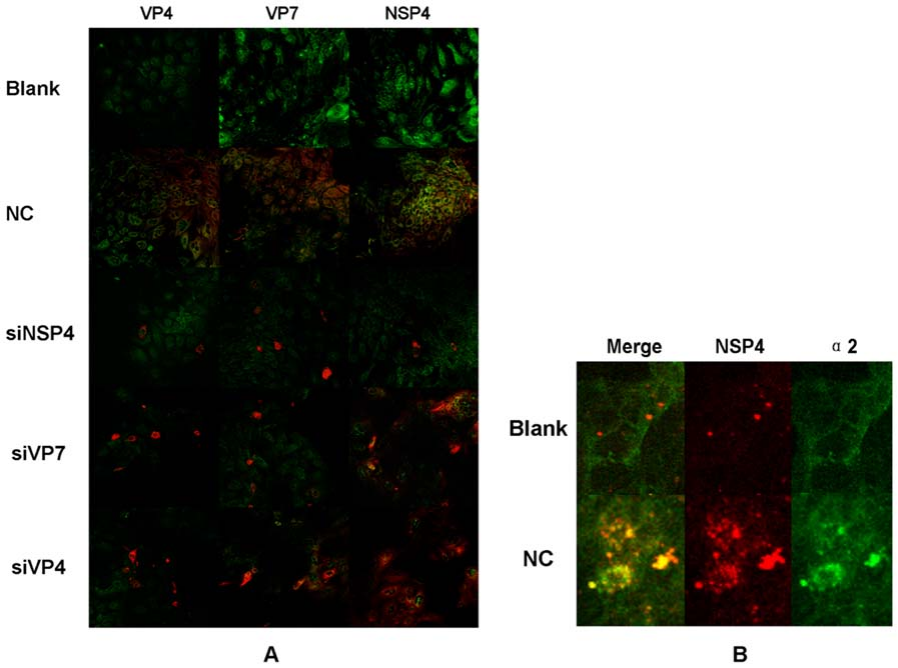
Immunofluorescence for rotavirus proteins and integrin α2. (A) All viral proteins were expressed in the cytoplasm of cultured extrahepatic biliary epithelia (EHBE). Decreased viral protein expression and less infected cells were evident in siNSP4 transfected EHBE. siVP7 transfection significantly reduced VP4 and VP7 expression while leaving NSP4 expression unchanged. siVP4 inhibited the expression of VP4, but the expression of the other 2 proteins was not influenced. Magnification, ×200. (B) NSP4 positive biliary epithelia had higher expression of integrin α2 compared to non-infected cells. Magnification, ×1000.

Immunofluoresent assay (IFA) showed that viral proteins were constantly expressed in the cytoplasm of EHBE. The distribution of NSP4, VP7 and VP4 was similar to each other, indicating a large amount of EHBE was productively infected in NC group. Significantly decreased number of EHBE contained translated VP4, VP7 and NSP4 after transfection of siNSP4. siVP7 transfection could down-regulate the translated products of VP4 and VP7 in EHBE after RRV infection, but positive staining of NSP4 in a large number of EHBE suggested most of cells could still express NSP4. siVP4 only silenced VP4 expression, while the other 2 proteins were still expressed (Figure 2A).

#### Integrin subunit α2 is increased in NSP4 positive cells

Integrin subunit α2 was expressed on all cultured EHBE. NSP4 positive cells had higher expression of integrin subunit α2 than that in the blank control (Figure 2B).

### In vivo studies

#### siRNA transfection decreases hepatobiliary injury and reduces the incidence of biliary atresia

**Figure 3:**
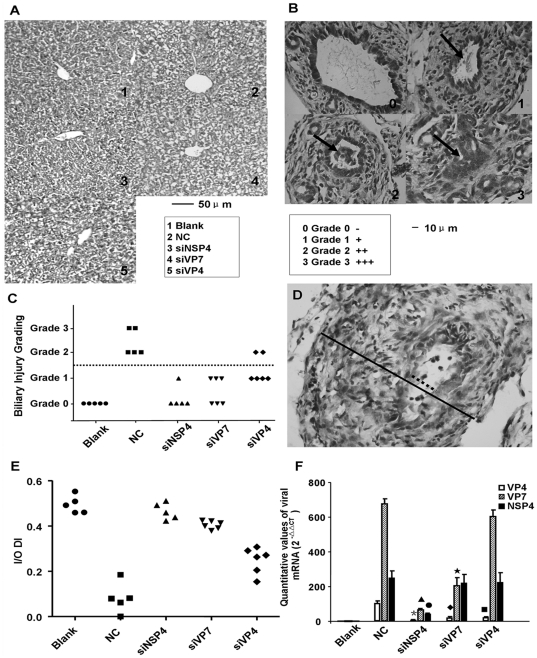
Hepatobiliary injury, incidence of biliary atresia and intraluminal rotavirus replication in the bile ducts. (A) Hematoxylin and eosin staining of livers. Ballooning degeneration and mononuclear cell infiltration were the basic pathologic changes in the liver of rotavirus infected mice in the NC group, but none suffered from hepatic cirrhosis. siNSP4 and siVP4 transfection decreased hepatic injury, but siVP7 transfected mice still suffered from significant hepatic injury. (B) Grading of extrahepatic bile duct injury. Grade 0: No obstruction, stenosis, necrotic epithelia or inflammatory cell infiltration. Grade 1: Mild stenosis and several inflammatory cells. Grade 2: Stenosis or obstruction caused by necrotic cells or inflammatory cells in bile duct lumens. Grade 3: Complete lumen obstruction. Black arrow pointed at the site of injury. (C) Summary of distribution of biliary injury grading. Biliary injury was significantly inhibited by siNSP4 (0/5), (*P*<0.05) compared to NC (5/5). Three of siVP7 transfected mice suffered mild biliary injury. Half of siVP4 transfected mice suffered BA. (D) Measurement of inner and outer diameters of bile ducts. The dashed line and black line respectively indicated the inner and outer diameters. (E) Summary of distribution of inner/outer diameter index (I/O DI). siRNA transfected mice had relatively higher index value (all *P*<0.05, compared to NC). siNSP4 had the highest I/O DI which was not significantly different from the blank controls (*P*>0.05). (F) Quantitative analysis of viral messenger RNA (mRNA) in bile ducts on 7 dpi using real-time reverse transcription polymerase chain reaction. siNSP4 decreased the level of all viral mRNAs (^*▴•^ *P*<0.05, compared to NC). siVP7 inhibited the transcription of VP7 and VP4 mRNAs (^♦★^ *P*<0.05, compared to NC). siVP4 only inhibited the mRNA expression of VP4 (^▪^ *P*<0.05, compared to NC).

Hematoxylin and eosin staining of the liver showed that siNSP4 and siVP4 transfection prevented hepatic injury (Figure 3A). According to Petersen C's murine model of BA [9], Grade 2 and 3 were considered as BA. siNSP4 transfection efficiently prevented extrahepatic bile ducts from rotavirus induced biliary atresia with the lowest incidence of BA (0/5). In the siVP7 group and siRNA targeting VP4 (siVP4) group, siRNA transfected mice also reduced the incidence of BA (0/6 and 2/6 respectively), but some mice still suffered relatively severe biliary injury in the 2 groups (Figure 3B and 3C). Moreover, measurement of the inner/outer diameter index (I/O DI) suggested that siNSP4 transfection could restore normal bile flow in the well preserved bile duct lumens (Figure 3D and 3E).

#### siRNA transfection decreases viral mRNA levels on day 7 post infection

All 3 rotavirus mRNAs were inhibited significantly by siNSP4. siVP7 and siVP4 transfection could not reduce the transcription of NSP4. siVP7 could silence VP7 mRNA and reduce the synthesis of VP4 mRNA. siVP4 could only significantly decrease the level of VP4 mRNA (Figure 3F).

#### Expression of viral proteins is decreased by siRNAs on mucosal layer of the bile ducts

**Figure 4:**
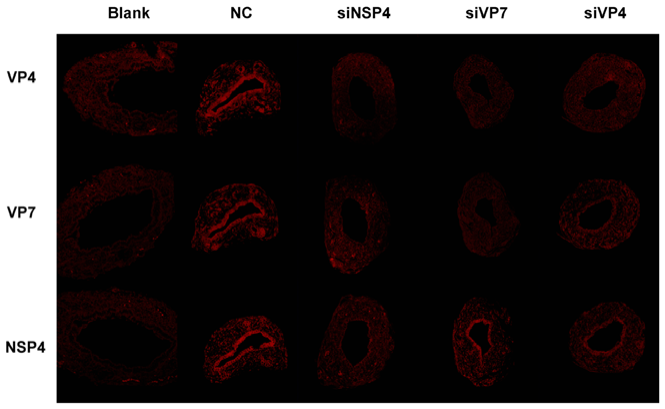
Immunofluorescent assay for viral protein expression in biliary epithelia of extrahepatic bile ducts on 7 dpi. Extrahepatic bile ducts infected by rotavirus expressed cytoplasmic viral proteins in the epithelial layer. siNSP4 transfection decreased the expression of NSP4, VP7 and VP4. In serial sections, NSP4 was positive but VP4 and VP7 were both negative in siVP7 transfected mice. siVP4 transfected mice had positive expression of VP7 and NSP4.

The mucosal layer was the major site for rotavirus infection in bile ducts. siNSP4 transfected mice had low expression of NSP4, VP7 and VP4 proteins. Moreover their bile duct lumens were not narrowed nor obstructed. Expressions of VP4 and VP7 were not detected in siVP7 transfected mice. In siVP4 protected mice, VP4 was negatively and VP7 was weakly positive. (Figure 4).

## Discussion

It has been over 30 years since the report of a model of reovirus induced BA in rodents [15]. Since then, there have been several advances in this model including the study of rotavirus, an important human pathogen [5]. Our previous findings indicate that rotavirus-induced biliary atresia is mediated by nuclear factor-kappaB [10] and abnormal activation of osteopontin inflammatory pathway in the liver [7]. Our newly established method for primary culture of biliary epithelia gives us a powerful means to study the relationship between rotavirus infection and biliary injury [16]. Rotavirus can infect biliary duct epithelial cells [17]. Therefore, EHBE is the presumptive target of rotavirus.

Previous data in bovine MA104 cells have shown that NSP4, VP7 and VP4 are essential for rotavirus replication and infection [11]. However, no research has ever verified whether NSP4, VP7 or VP4 protein are important in damaging EHBE and consequently causing BA. Based on our previous findings, we conducted a study of gene silencing to clarify the relationship between these three rotavirus proteins and rotavirus-induced biliary injury.

In this study, we show that NSP4 elimination can significantly decrease the quantity of TLPs and DLPs in EHBE. This suggests that very few mature viral particles are assembled. We detected the mRNA and protein levels of NSP4, VP7 and VP4, because VP4 and VP7 are also essential for TLP assembly. The results show that siRNA knockdown of NSP4 inhibits the synthesis of VP7 and VP4. This indicates that loss of function of NSP4 inhibits the synthesis of VP7 and VP4. [17], [18] Furthermore, NSP4 co-localizes with integrin α2 on the surface of rotavirus-infected mucosal epithelia in the bile ducts, indicating that NSP4 up-regulates the expression of integrin α2.

The incidence of murine BA, as reported in several studies, ranges from 50% to 80% [18], [19], [20]. In this study, we semi-quantitatively measured I/O DI, which provides a reliable assessment of the severity of biliary obstruction. All of the negative control (NC) mice developed BA, whereas mice treated with siNSP4 were protected from severe biliary injury. In addition, along with the inhibition of NSP4, the level of the other two viral mRNAs we assayed was markedly down regulated in the bile ducts. From both the in vitro and in vivo studies, we obtained similar results. Thus, the loss of NSP4 inhibited other viral mRNA and protein expression, as well as decreased epithelial injury on cultured EHBE and the mucosal layer of extrahepatic bile ducts. As a result, the incidence of biliary atresia decreased significantly.

In siVP7 transfected EHBE, increased proportion of DLPs is expected, because VP7 forms the outermost protein layer of rotavirus [12]. Administration of siVP7 decreased VP4 mRNA and protein level, but had no effect on NSP4. Mice in this group still have hepatobiliary injury. VP7 loss of function may be compensated by other pathways which may be directed by NSP4 or other viral proteins. This indicates that VP7 may not be essential in the pathogenesis of BA, but it may have an important role in modulating the maturity of rotavirus in EHBE. We postulate that VP7 may function in the intermediate step in the pathogenesis of BA, but we need to perform more investigations to clarify how it reacts with other viral proteins and EHBE.

VP4 loss of function can neither prevent EHBE from rotavirus-induced biliary injury, nor can it achieve prevention of BA. However, when severe ballooning degeneration is noted in the infected mice, siVP4 transfected mice get mild ballooning degrneration. This is probably because VP4 is important in cell attachment, penetration, and virulence determination [21]. Moreover, siVP4 treatment results in virus particles lacking the VP4 spikes, thereby decreasing TLPs. Virus particles formed would not spread to uninfected EHBE. However, damage to epithelium still occurs, perhaps due to the continued production of NSP4 and VP7, as shown above. Thus, while VP4 may play overall role in rotavirus pathogenesis in the intestine, it is not a major determinant of rotavirus induced BA.

Our data suggests that the non-structural protein of rotavirus plays a major role in the pathogenesis of BA in a murine model. There is an interaction of NSP4 with two other known determinants for pathogenic interaction of rotavirus. However, these proteins appear to play a less important role, based upon the partial protection afforded with knock down of expression. The exact mechanism for NSP4 inducing biliary disease is not clear. Several models can be advanced, including affects on secondary host proteins, such as integrin a2, or immune response. Further work is needed to delineate the precise role of NSP4 in BA disease.

## Materials and Methods

### Ethics statement

All of the studies were carried out in accordance with Communities Council Directive for care of laboratory animals in an AAALAC-accredited facility following approval of study design (Permit Number 2009-AR0288) by the Institutional Animal Care and Use of the Committee (IACUC) at Tongji Medical College (Wuhan, China). Veterinarians skilled in the healthcare and maintenance of rodents supervised animal care. Reasonable efforts were made to minimize suffering of animals. The use of animals was minimized by using an experimental design permitting statistically-significant changes to be demonstrated with the smallest number of animals per group and the smallest number of groups, which was consistent with scientific rigour.

### Cell and virus preparation

Rhesus monkey kidney epithelial cell line (MA104) was provided by Dr. Yuanhong Wang (Center of Disease Control, Wuhan, China). RRV strain mmu18006 was obtained from Dr. Greg M. Tiao (Pediatric Surgery Division and Liver Care Center, Cincinnati Children's Hospital Medical Center, USA). RRV was propagated on MA104, Dulbecco's Modified Eagle Medium (DMEM, GIBCO, Grand Island, N.Y., USA) with trypsin (3 µg/ml, Sigma, St. Louis, M.A., USA), 1% penicillin and 1% phytomycin. When cytopathic effect (CPE) percentage reached to 80%, RRV was harvested and its titer was determined by the plaque forming assay. The RRV titer of supernatant was 2.0×10^6^ Plaque Forming Unit per ml.

### siRNAs and Primers

The siRNAs targeting RRV genes were produced by Guangzhou RIBOBIO Corporation, Guangdong, China. The siRNA sequences and mRNA targets were listed in Table 2. siRNA Lamin A/C (AAC UGG ACU UCC AGA AGA ACA) served as the control siRNA [12]. siRNAs and primers were designed and based on the rhesus rotavirus mRNA sequences of NSP4 (Genebank Accession Number EU636933), VP7 (Accession Number AF295303.1) and VP4 (Accession Number AY033150).

**Table 2:** siRNA targets and sequences against NSP4, VP7 and VP4.

	siNSP4	siVP7	siVP4
**Gene Target**	GGCCTCGGTTCCAACCATG	CTAGAATGATGGACTTTAT	CCAGCAAACTATCAATATA
**Positive-sense strain**	5′GGCCUCGGUUCCAACCAUG dTdT 3′	5′CUAGAAUGAUGGACUUUAU dTdT3′	5′CCAGCAAACUAUCAAUAUAdTdT3′
**Anti-sense strain**	3′dTdT CCGGAGCCAAGGUUGGUAC5′	3′dTdT GAUCUUACUACCUGAAAUA5′	3′dTdTGGUCGUUUGAUAGUUAUAU5′

### In vitro studies

#### Isolation and culture of EHBE

The primary EHBE were cultured based on the method described in our previous work [16]. Briefly, the extrahepatic bile ducts of adult Balb/C mice were removed and transferred into DMEM/HamF12 medium (GIBCO, Grand Island, N.Y., USA). The bile ducts were cut into fragments with the diameter less than 0.5 mm and digested by 0.25% trypsin and DNase I (20 IU/ml, Invitrogen, Carlsbad, C.A., USA) for 10 mins at 37°C. Subsequently, collagenase IV (200 U/ml, Invitrogen, Carlsbad, CA, USA) was added and incubated for 30 mins at 37°C. After centrifugation, the detached cells were re-suspended and separated from the undigested tissues by filtration. The cell density was adjusted to 1×10^6^/ml. The cells were seeded in plastic culture-flasks containing DMEM/HamF12 with 10% fetal bovine serum and epidermal growth factor (10 ng/ml, PeproTech, Rochy Hill, N.J., USA). On the 4th day of culturing, cultured EHBE were identified by cytokeratin-19 immunocytochemical staining and MTT cell proliferation assay [16].

#### SiRNA transfection and RRV infection

After the EHBE were washed with PBS, 2 ml of Opti-MEM-I (Invitrogen, Carsbad, C.A., USA) was added. After 2 h, Opti-MEM-I was removed. The transfection mixture consisting of 0.49 ml of Opti-MEM-I, 0.1 nmol of siRNA and 5 ul of lipofectin^TM^2000 (Invitrogen, Carlsbad, C.A., USA) was added. Six hours later, 1.5 ml of DMEM with 10% fetal bovine serum was added. At 24 h post-transfection, the transfection mixture was removed. The EHBE was incubated with 500 µl of RRV supernatant at 37°C. The EHBE in the blank control group were transfected with transfection mixture with siRNA Lamin A/C, with no RRV infection. The EHBE the NC group were infected with RRV and transfected with siRNA Lamin A/C. The EHBE in the siRNA groups were transfected with corresponding siRNA and then infected with RRV.

#### CPE determination

According to the method of titer determination by Landau SM [22], at 24 hours post inoculation, CPE percentage in 6 random vision fields (×100 magnification) was determined and expressed as mean±SD from 3 independent experiments.

#### Ultrastructure of EHBE and virus quantification

Based on the modified method of Esparza J, et al [23], the cell suspension was centrifuged for 10 min at 1000 rpm. The cell pellet was washed with PBS, fixed in 2.5% glutaraldehyde overnight at 4°C and postfixed in 2% OsO_4_ for 2 h and dehydrated. Fixed cell masses were embedded in Epon-812 and made into ultra-thin sections with the thickness of 100 nm. The sections were further stained with uranyl acetate and lead citrate. Sections were observed under transmission electron microscope (FEI-Tecnai G2-12-type, Holland). Viral particles in 6 random vision fields were counted. The number of viral particles was expressed as mean±SD from 3 independent experiments.

#### Reverse transcription polymerase chain reaction

For the evaluation of mRNA of NSP4, VP7 and VP4 in the cultured EHBE, total RNA was isolated from EHBE with the Trizol reagent, and 2 µl of total RNA was reverse transcribed to synthesize cDNA with a ReverTra Ace-α- kit (TOYOBO, FSK-100, Japan). Each PCR reaction containing 1 µl of cDNA, 2.5 ul of 10×PCR Buffer, 0.25 µl of dNTP (10 mmol/L), 0.5 µl of primers (5 pmol/µl, Table 3), 0.25 µl of rTaq DNA polymerase and 20.5 µl of deionized water was heated at 95°C for 5 mins, followed by 30 cycles consisting of 95°C for 25 s, renaturation (at 58°C for β-actin and NSP4, at 60°C for VP4 and VP7) for 25 s and 72°C for 30 s. After cycles accomplished, the amplification products were maintained at 72°C for 3 mins. All tested samples were run in triplicate. The PCR products were subjected to electrophoresis on 1.5% agrose gels containing ethidium bromide. Gel-Pro Analyzer (Version 3.0) was used to analyze the relative levels of mRNA. The relative value of target mRNA/β-actin in the NC group was defined as 100%, and relative value in each group was expressed as mean±SD from 3 independent experiments.

**Table 3:** Primers for mRNA of NSP4, VP7, VP4 and β-actin.

	NSP4	VP7	VP4	β-actin
**First Primer 5**′ **to 3**′	TTTCCATACATTGCTTCTGTCCT	CTGACGTTGTCGATGGCGTG	GAAGCGGGAACAGATGGAAGA	CCGTGAAAAGATGACCCAG
**Second Primer 5**′ **to 3**′	CAACTCTGTCCATTTGCCTTTC	CAGTTTGTGGTGCTGTGGTTGG	TGGCTGAGATGACCGGAGAGT	TAGCCACGCTCGGTCAGG

#### IFA of rotavirus protein in cultured EHBE

Primary antibodies (mouse IgG, 2 µl/ml) for detecting NSP4, VP7 or VP4 were kindly provided by Prof. Harry Greenberg (Stanford University School of Medicine, Stanford, USA). Integrin subunit α2 on the EHBE was detected by FITC-labeled Hamster-anti-mouse integrin subunit α2 (CD49b) antibody (Biolegend, San Diego, C.A., USA), in order to label all EHBE. The EHBE were fixed with 4% paraformaldehyde for 30 mins at 37°C. Nonspecific reactions were blocked with normal goat serum at 37°C for 30 mins. After incubated with primary antibodies at 4°C overnight, EHBE was incubated at 37°C for 2 h with Rhodamine-conjugated goat-anti-mouse secondary antibody (Santa Cruz, San Diego, C.A., USA) and analyzed with a confocal fluorescence microscope (FV500, Olympus, Japan).

### In vivo studies

#### Animals, siRNA transfection and RRV infection

In the first 6 h of life after birth, 5 pups in the siNSP4 group, 6 pups in the siVP7 group and 6 pups in the siVP4 group underwent intraperitoneal injection of transfection mixture consisting of siRNA (0.15 nmol/g of bodyweight) dissolved in 50 µl of *in vivo-jet* PEI^TM^ (201-10G, Polyplus transfection Inc, C.A., USA) with the N/P ratio of 6 according to the instruction. The pups in the blank (N = 5) or the NC group (N = 5) were subjected to siRNA Lamin A/C transfection. Six hours later, all pups were injected with 50 µl of RRV supernatant except the blank group. After RRV infection, all pups were monitored for 14 days. On 7 dpi, half of the pups in each group were sacrificed, and their bile ducts were used for mRNA and protein detection. The rest were euthanized on 14 dpi, and their extrahepatic bile ducts and livers were harvested.

#### Histopathology

Formalin fixed, paraffin-embedded bile duct and liver sections were stained with hematoxylin and eosin. The I/O DI, representing the value of bile duct diameter divided by lumen diameter, was calculated and expressed as mean±SD.

#### Real-time reverse transcription polymerase chain reaction

Total RNA was isolated from EHBE with the Trizol reagent, and 2 µl of total RNA was reverse transcribed to synthesize cDNA with a ReverTra Ace-α- kit (TOYOBO, FSK-100, Japan). To carry out relative quantification, real-time RT-PCR was performed for measurements of NSP4, VP7 and VP4 mRNAs according to a standard protocol using the SYBR Green PCR Master Mix (Qiagen, C.A. USA) and ABI-Prism 7700 Sequence detection system (Applied Biosystems, Tokyo, Japan). Each PCR reaction contained 12.5 µl of SYBR-Green mix, 2.5 µl of plus-solution, 2 µl of primers (5 pmol/µl), 8 µl of ddH_2_O and 2.5 µl of cDNA. After the cDNA were preheated at 50°C for 2 mins and heated at 95°C for 10 mins, it was followed by 30 cycles consisting of 95°C for 15 s, renaturation (at 58°C for β-actin and NSP4, at 60°C for VP4 and VP7) for 15 s and 72°C for 45 s. After these cycles, the amplification products were maintained at 72°C for 10 mins. All tested samples were run in triplicate. The value of 2^−△△CT^ was expressed as mean±SD from 3 independent experiments.

#### IFA of rotavirus proteins in mucosal layer of biliary epithelia

Serial-sections were made for IFA. After nonspecific reaction was blocked, primary antibody of NSP4, VP7 or VP4 was incubated at 4°C overnight. Rhodamine-conjugated goat-anti-mouse secondary antibody (Santa cruz, San Diego, C.A., USA) was then incubated at 37°C for 1 h. The slides were observed with a confocal fluorescence microscope (FV500, Olympus, Japan).

#### Statistical analysis

Analysis of Variance (ANOVA) was used to compare mean values. Fisher's exact test was used to compare the incidence of BA. All data was analyzed with *SPSS* 11.0 (*SPSS*, Chicago, III). *P*<0.05 was considered statistically significant.
